# Automation of the Radiosynthesis of Six Different ^18^F-labeled radiotracers on the AllinOne

**DOI:** 10.1186/s41181-016-0018-0

**Published:** 2016-10-10

**Authors:** Shihong Li, Alexander Schmitz, Hsiaoju Lee, Robert H. Mach

**Affiliations:** Department of Radiology, University of Pennsylvania, Philadelphia, PA USA

**Keywords:** PET, Radiotracer, Automation, AllinOne, HPLC, SPE, ISO-1, FTP, FTT, F-Gln

## Abstract

**Background:**

Fast implementation of positron emission tomography (PET) into clinical and preclinical studies highly demands automated synthesis for the preparation of PET radiopharmaceuticals in a safe and reproducible manner. The aim of this study was to develop automated synthesis methods for these six ^18^F-labeled radiopharmaceuticals produced on a routine basis at the University of Pennsylvania using the AllinOne synthesis module.

**Results:**

The development of automated syntheses with varying complexity was accomplished including HPLC purification, SPE procedures and final formulation with sterile filtration. The six radiopharmaceuticals were obtained in high yield and high specific activity with full automation on the AllinOne synthesis module under current good manufacturing practice (cGMP) guidelines.

**Conclusion:**

The study demonstrates the versatility of this synthesis module for the preparation of a wide variety of ^18^F-labeled radiopharmaceuticals for PET imaging studies.

## Background

Positron emission tomography (PET) facilities have recently been growing exponentially as PET is an especially sensitive molecular imaging technique quantitatively measuring tracers in nano- to picomolar range in comparison with other modalities like magnetic resonance imaging (MRI) or computerized tomography (CT). The main limitation of PET is the short half-lives of the radionuclides used in the development of PET radiotracers. Among the most popular positron-emitting radionuclides, short half-life radionuclides like oxygen-15 and nitrogen-13 are used for brain perfusion studies ([^15^O]H_2_O) and heart perfusion studies ([^13^N]NH_3_) respectively (Bergmann et al., 1989; Grüner et al., 2011; Muzik et al., 1993). The 20.4 min half-life of carbon-11 and the rapid production of reactive intermediates such as [^11^C]methyl iodide and [^11^C]methyltriflate have facilitated the generation of ^11^C-labeled radiotracers for a variety of imaging applications. However, the 110 min half-life of fluorine-18 has firmly established itself as the radionuclide of choice for imaging applications since it allows for longer data acquisition for dynamic imaging studies and high count rates for metabolite analyses which are often required for quantitative PET imaging studies. In addition, fluorine-18 has a relatively low energy (maximum 0.635 MeV) and thus the emitted positron has a short mean range (2.39 mm in water) (Vallabhajosula, 2009).

The methods for fluorine-18 labeling have been greatly improved in the last 30 years, largely due to recent advances in organic fluorination chemistry (Brooks et al., 2014; Jacobson et al., 2015; Kamlet et al., 2013; Li and Conti, 2010; Mach and Schwarz, 2010; Rotstein et al., 2014). Therefore, the utilization of ^18^F-labeled radiopharmaceuticals for human studies has expanded greatly in various areas of clinical research, such as cancer, neurological disorders, and cardiac diseases. The key requirements for the synthesis of radiopharmaceuticals used for clinical PET studies are reproducibility, reliability and short synthesis time. Full automation is also important, especially for general-purpose nucleophilic radiofluorination reactions. The historical background and evolution of automated synthesis for radiopharmaceuticals has been described in a number of excellent reviews (I Sachinidis et al., 2010; Krasikova, 2007; Preshlock et al., 2016; Shao et al., 2011; Welch and Redvanly, 2003), and a guidance on current good radiopharmaceuticals using automated modules was created in Europe in 2014 (Aerts et al., 2014). There are a number of commercially-available automated synthesis modules designed for [^18^F]FDG radiosynthesis, and these have been modified for the synthesis of other ^18^F-labeled radiotracers. These automated modules are designed to conduct 2–3 organic reactions, followed by either resin-based SPE and/or HPLC purification. Examples of commercial modules include FASTLab^TM^, TracerLab™ FXFN series (GE Healthcare), E-Z modules (Eckert & Ziegler modular lab) and Explora® series (Siemens Healthcare). Recently, the AllinOne, an automated synthesizer by Trasis, was developed as a universal GMP-compliant synthesis module for radiolabeling of radiotracers with short half-life radionuclides. This module is described as being versatile and capable of handling complex chemistry. The instrument can be broken down to the chemistry module, purification module and reformulation module. Disposable cassettes, reagents and components are used to ease the burden on cleaning. Successful production with high yield and high specific activity to the following compound, such as [^18^F]F-DOPA, [^18^F]FDG, [^18^F]MPPF, [^18^F]FES, [^18^F]F-MISO, [^18^F]FET, and [^18^F]FLT has been reported (Otabashi et al., 2015). These radiotracers were developed many years ago and have been used on either an intermediate to widespread basis in PET imaging studies.

Although [^18^F]FDG is an effective tumor-imaging agent for diagnosis, staging, restaging and monitoring various malignant conditions, its utilization has several well-known limitations (Lind et al., 2004; Liu et al., 2014; Lubezky et al., 2007; Pery et al., 2010; Suzuki et al., 2008), such as non FDG-avid tumors (e.g. prostate cancer) and non-cancerous FDG-avid tissue (e.g. inflammatory tissue). Therefore, the introduction of new PET radiopharmaceuticals capable of filling these gaps is clearly needed. Recently, glutamine was suggested as alternative source of metabolic energy for tumor cells (Wise et al., 2008). Glutaminolysis, especially in myc-overexpressing cells, plays a significant role in tumor growth and metabolism. Fluorine-substituted glutamine analogs have been particularly useful in PET imaging studies, with [^18^F]-(*2S,4R*)4-Fluoroglutamine ([^18^F]F-Gln) being the most promising metabolic tracer for imaging glutamine metabolism (Lieberman et al., 2011; Ploessl et al., 2012; Qu et al., 2010).

The development of small molecules targeting proteins overexpressed in cancer cells is another application that is gaining and has increased importance in oncologic imaging studies. For example, the sigma-2 receptor is overexpressed in most human and murine tumors and has been proposed as a biomarker for imaging tumor cell proliferation (Mach et al., 1997). The selective sigma-2 receptor radiotracer [^18^F]ISO-1 was evaluated in Balb/c female mice bearing EMT-6 mammary allografts, and an initial evaluation of this radiotracer in patients with either lymphoma, breast or head & neck cancer was recently reported (Tu et al., 2007). The high correlation of tumor-to-muscle ratio to *Ki*-67 scores indicates that [^18^F]ISO-1 may provide a novel method for imaging the proliferative status of solid tumors. [^18^F]ISO-1 may be capable of stratifying patients into groups of high or low proliferative status, which is expected to be useful in selecting patients who are likely to respond to cell cycle specific chemotherapeutics (Dehdashti et al., 2013). Meanwhile, poly ADP-ribose polymerase-1 (PARP-1) is critical to DNA repair and PARP-1 inhibition has been demonstrated as an effective method for inducing synthetic lethality in cancers depending on PARP-1 activity for survival. The uptake of the PARP-1 radiotracer, [^18^F]FluorThanatrace ([^18^F]FTT), was found to correlate with PARP-1 expression (Edmonds et al., 2016; Zhou et al., 2014). Quantifying tumoral PARP-1 activity with PET should be particularly useful for occupancy studies aimed at determining the optimal dose of a PARP-1 inhibitor generating an optimal therapeutic response.

In addition to radiopharmaceuticals used for cancer imaging, a number of receptor-based radiopharmaceuticals have been developed to study a wide variety of central nervous system (CNS) disorders including Alzheimer’s disease (AD), Parkinson’s disease (PD), depression and drug addiction (Sokoloff et al., 1990). [^18^F]Flubatine, short for (-)- [^18^F]flubatine, has been used to image nicotinic acetylcholine receptors (nAChRs) since a dysfunction of the cholinergic neurotransmitter system is one factor contributing to cognitive decline in neurodegenerative disorders such as Alzheimer’s disease (Bois et al., 2015; Gallezot et al., 2014; Hockley et al., 2013; Wu et al., 2010). [^18^F]Fallypride has been developed to study extrastriatal dopamine D_2_ receptor expression in a number of neuropsychiatric disorders (Mukherjee et al., 1995; Mukherjee et al., 1997; Mukherjee et al., 2002; Riccardi et al., 2006; Slifstein et al., 2010). And finally, our group has developed the first high affinity, D_3_–selective PET ligand, [^18^F]Fluortriopride ([^18^F]FTP), which has demonstrated feasibility for imaging D_3_ receptors in non-human primate brain, following depletion of endogenous dopamine (Mach et al., 2011; Tu et al., 2011).

**Fig. 1:**
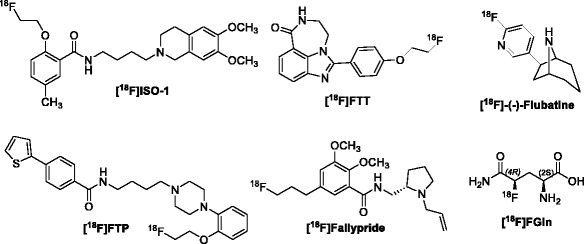
Structures of six radiotracers synthesized on the AllinOne module

Although straightforward in theory, the adaptation of structurally-diverse PET radiotracer syntheses onto an automated system requires considerable expertise in the field of organic chemistry and chemical engineering. In the current report, we describe the fully automated syntheses on the AllinOne module of the six radiotracers described above (Fig. 1). The automation process includes radiolabeling, purification, formulation and system cleaning. To date, [^18^F]ISO-1, [^18^F]FTP and [^18^F]FTT have been reliably produced for use in phase 0 clinical studies, while [^18^F]F-Gln, [^18^F]flubatine and [^18^F]fallypride were used for microPET, cell uptake and metabolism studies.

## Methods

### Materials for synthesis and analysis

[^18^F]F-Gln tosylate precursor, (2S, 4R)-tert-butyl-2-(tert-butoxycarbonylamino)-5-oxo-4-(tosyloxy)-5-(2, 4, 6-trimethoxybenzylamino)pentanoate, and reference standard were obtained from MSKCCC (New York, USA) or from Dr. Hank Kung. [^18^F]ISO-1 reference standard and its mesylate precursor, 2-(2-((4-(6,7-dimethoxy-3,4-dihydroisoquinolin-2(1H)-yl)butyl)carbamoyl)-4-methylphenoxy)ethyl methanesulfonate,; [^18^F]FTT reference standard and its precursor, 2-(4-(6-oxo-6,7,8,9-tetrahydro-2,7,9a-triazabenzo[cd]azulen-1-yl)phenoxy)ethyl 4-methylbenzenesulfonate; and [^18^F]FTP reference standard and its precursor 2-(2-(4-(4-(4-(thiophen-2-yl)benzamido)butyl)piperazin-1-yl)phenoxy)ethyl methanesulfonate, were made in our lab under GLP conditions. Cryptand (as shown as K222 in the following text), [^18^F]fallypride reference standard and its tosylate precursor, and [^18^F]flubatine reference standard and its iodide precursor were all obtained from ABX (Radeberg, German).

Acetonitrile (MeCN, anhydrous for synthesis; HPLC grade for purification and analysis), dimethylformamide (DMF, anhydrous, dried over molecular sieves (4 Å)), dimethyl sulfoxide (DMSO, anhydrous, dried over molecular sieves (4 Å)), methanol (MeOH, HPLC grade), potassium carbonate (K_2_CO_3_, ACS grade), ammonium formate (NH_4_HCO_2_, ACS grade), ammonium bicarbonate (NH_4_HCO_3_, ACS grade) were purchased from Sigma-Aldrich (St Louis, USA). Water (DIUF grade) and trifluoroacetic acid (TFA, anhydrous, HPLC grade) were purchased from Fisher Scientific (Pittsburgh, USA). Cartridges used for solid phase extractions, such as Sep-Pak QMA Carb Cartridges, Sep-Pak C18 Plus Cartridges, Sep-Pak ^t^C18 Cartridges, Alumina N Plus Cartridges, Sep-Pak Dry Sodium Sulfate Plus Long Cartridges, and Sep-Pak Silica Plus Long Cartridges, were purchased from Waters (Milford, USA). Sterile water for injection (250 mL bags) was purchased from B. Braun (Melsungen, Germany). Sterile normal saline (0.9 % w/v) was purchased from Hospira (Lake Forest, USA) and ethanol (200 proof, USP grade) was from Decon Labs (King of Prussia, USA). ^18^O water (>97 %) was purchased from Huayi Isotopes (Changshu, China) or ABX (Radeberg, Germany). Low-protein binding 0.2 μm sterile filters, Millex® FG filters, were obtained from Millipore (Bedford, USA) and 0.45 μm nylon filters, Whatman Puradisc®, were purchased from GE Healthcare (Marlborough, USA). All other chemicals and solvents for radiosyntheses were obtained from Sigma-Aldrich or Fisher Scientific. These chemicals are at least ACS grade and used without further purification. AllinOne synthesis module and all consumable parts were purchased from TRASIS (Ans, Belgium).

### Instrumentation for general procedures

**Fig. 2:**
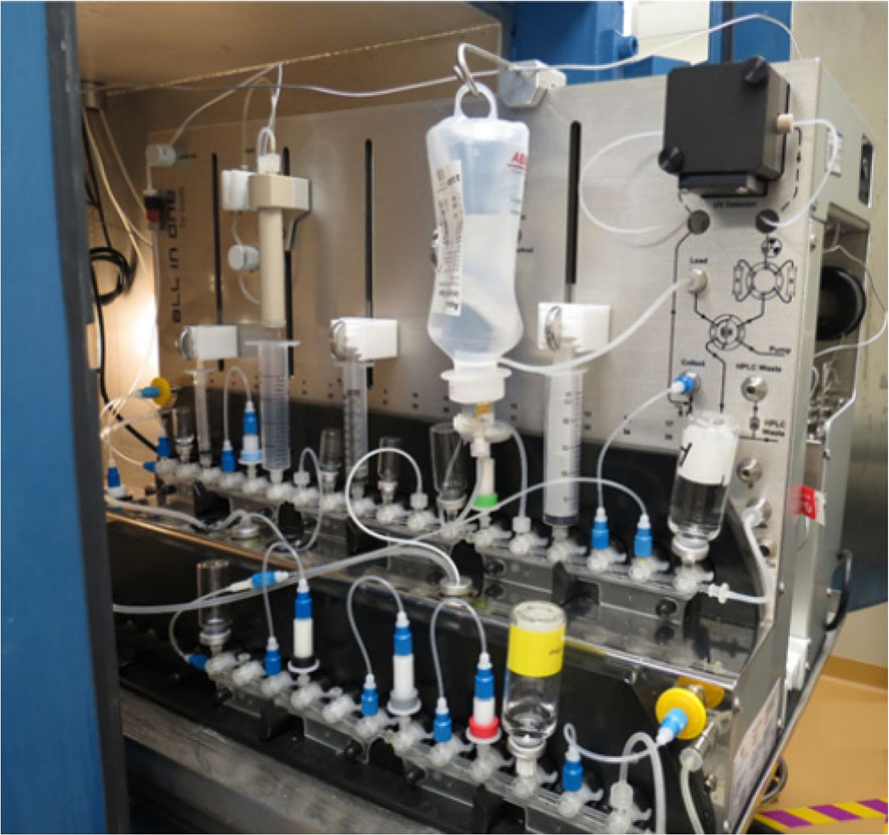
AllinOne synthesis module with mounted disposable kits and reagents for [^18^F]FGln

**Fig. 3:**
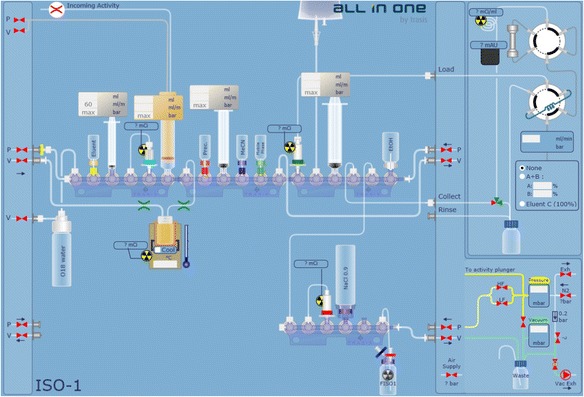
AllinOne user software interface for one-vessel reaction ([^18^F]ISO-1)

**Fig. 4:**
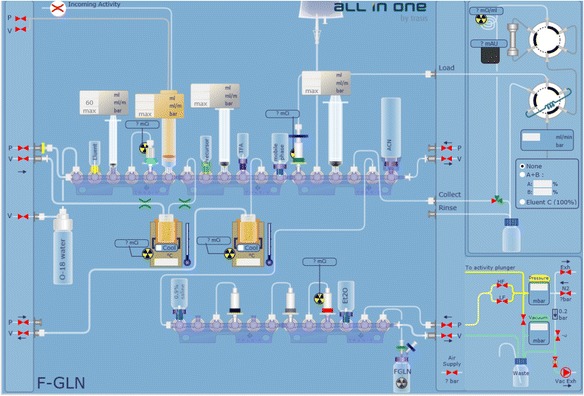
AllinOne user software interface for two-vessel reaction ([^18^F]F-Gln)

The AllinOne synthesis module is a thirty-actuator version (with option for 18–36 actuators), with two reaction vessels, and a built-in HPLC system with UV detector and radioactivity detector. The cassette is structured around zero dead volume three-way valve manifolds. Each manifold contains six-valve positions and can be extended or removed freely. Each position can be assigned to reagent vials, SPE cartridges, syringes or others functions. Those connections are mostly through spikes and tubing. All components are compatible with the most aggressive acids, bases and solvents. Generally speaking, we use the first six valves for F-18 drying, the second set of valves for first-step of radiolabeling and the third set for second-step reaction and/or sample preparation prior to HPLC purification. Product formulation is generally accomplished by the last set of valves. Figure 2 displays the module with loaded cassette and reagents for [^18^F]F-Gln. Synthesis protocols are generated using the accompanying user-friendly software that allows the user to string together all sequences for each operational step, such as drying, radiolabeling and formulating, to one synthesis method. Figure 3 shows the user interface for [^18^F]ISO-1 and Fig. 4 shows the user interface for [^18^F]F-Gln. After synthesis, the reaction module is left for decay until the next day due to the concern of radiation exposure. It is, however, possible to develop a cleaning protocol to rinse off the residual radioactivity from the cassette and vials if the need for using the module a second time arises.

Preconditioning is necessary for some cartridges before synthesis. Silica Plus Cartridges and Dry Sodium Sulfate Plus Long Cartridges were used as received. QMA Carb Cartridges were treated with 10 mL 0.2 M K_2_CO_3_ solution followed by 20 mL of water. C18 Plus and ^t^C18 Cartridges were preconditioned with 1 mL ethanol, and followed by rinse with 10 mL water. Alumina-N Plus Cartridges were treated with 10 mL water. All conditioned cartridges were kept wet unless otherwise specified. Typically, it takes thirty minutes to one hour to set up the module before receiving radioactivity to start the radiosynthesis. After loading the appropriate labeling program, the “machine check” sequence is performed to ensure module readiness including nitrogen pressure, compressed air pressure, vacuum, heating, cooling, and movement of syringe and manifold actuators. After kit installation, a “kit check” sequence is initiated to assure that the kit has been properly installed. After reagents are loaded, the module is ready to receive radioactivity for radiosynthesis.

No-carrier-added [^18^F]F^-^ was produced with the IBA cyclotron, Cyclone 18/9 (Louvain-La-Neuve, Belgium), via the ^18^O (p, n) ^18^F reaction. The initial radiolabeling was performed manually with a small amount radioactivity, 30–50 mCi, in a lead-shielded hot cell. When a desirable radiolabeling condition was found, the procedures were programmed into the AllinOne with the standard pressure and vacuum condition for drying [^18^F]F^-^ and radiolabeling temperature and duration adapted from the manual radiolabling. The program would be tested initially without radioactivity by running it with intended reagents/solvents but without fluoride source. Afterward, a small amount of starting radioactivity, 30–50 mCi, would be used to test whether the parameters from manual labeling can be translated directly to automated syntheses. Adjustment on the [^18^F]F^-^ drying and the radiolabeling temperature and duration are the two most common parameters that require optimization before the radiolabeling processes are set.

**Fig. 5:**
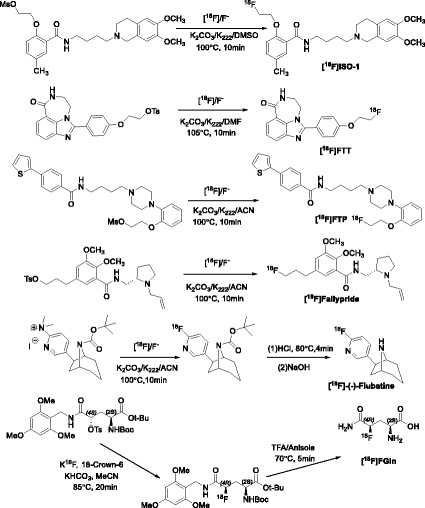
Scheme for the radiosyntheses of the six radiotracers

The radiolabeling was performed in a 6 mL flat-bottom glass vial with silicone crimp-cap with 2 tubing inlets. Therefore, for all the radiotracers that we adapted to the AllinOne module, the volume of radiolabeling solvent has to be adjusted to be at least 0.6 mL. To concentrate the collected radiotracer fraction after semi-preparative HPLC purification, C18 Sep-Pak cartridges were also incorporated into the automation process. Some adjustment on the semi-prep HPLC condition was also needed to provide better separation. These are the extent of optimization for [^18^F]fallypride and [^18^F]flubatine as we currently only use these two radiotracers for pre-clinical studies. Additional optimization details are described in Radiolabeling methods for [^18^F]F-Gln, [^18^F]ISO-1, [^18^F]FTP, and [^18^F]FTT. The final reaction conditions are listed in Fig. 5. Generally speaking, the precursor was heated in an aprotic solvent (DMSO, DMF or MeCN) with the corresponding phase-transfer reagent (K222 or 18-crown-6) for a predetermined time, followed by deprotection step when required, and then quenched with the HPLC mobile phase for purification.

Purification of all [^18^F]-labeled PET tracers included semi-preparative HPLC purification and solid phase extraction. Unreacted [^18^F]F^-^ was removed from the reaction mixture via Alumina N Plus cartridge prior to loading to HPLC. HPLC purification was performed with the built-in semi-preparative HPLC system equipped with a radioactivity detector, Smartline UV detector 200 (Knauer, Berlin, Germany) and a HPLC pump P4.1.5 (Knauer, Berlin, Germany). For semi-preparative HPLC purification columns, Luna® C18 column, 5 μm, 100 Å, 250 × 10 mm, was purchased from Phenomenex® (Torrance, USA); ZORBAX StableBond SB-C18 column, 5 μm 80 Å, 100 × 9.4 mm, was purchased from Agilent (Santa Clara, USA); SunFire C18 semi-preparative column, 5 μm, 250 × 10 mm, was purchased from Waters (Milford, USA).

Analytical HPLC was performed with a Waters Alliance e2695 HPLC system (Milford, USA) equipped with 2489 UV/VIS detector and photomultiplier scintillation radio-detector (Eckert&Ziegler, Berlin Germany). Zorbax XDB-C18, 5 μm 80 Å 150 × 4.6 mm, column (Agilent, Santa Clara, USA) was used for chemical and radiochemical purity testing for [^18^F]ISO-1, [^18^F]FTP, [^18^F]FTT, [^18^F]flubatine and [^18^F]fallypride. Phenomenex Chirex® 3126 (D)-penicillin column, 5 μm 250 × 4.6 mm (Santa Clara, USA)) and Sigma-Aldrich Astec® CHIROBIOTIC™ T Chiral column, 5 μm 250 × 4.6 mm (St Louis, USA) were used to confirm the enantiomeric of [^18^F]F-Gln. Residual solvent analysis was performed using an Agilent gas chromatography (GC) system, 6890 or 7890 series. All GCs and HPLCs were controlled with Waters Empower software.

### Radiolabeling methods

The labeling methods are listed in Table 1. Most of the processes utilize a one-step reaction, involving only one reaction vessel and a solid phase cartridge enrichment after HPLC purification. Although the synthesis of [^18^F]flubatine is a two-step reaction, the process still only involves one reaction vessel. The synthesis of [^18^F]F-Gln represents an exception. That is, the process is complicated, consisting of a two-step, two reaction-vessel followed by a multiple purification procedure including HPLC purification of the intermediate radiolabeled compound.

**Table 1:** Labeling methods and yields

Name of the radiotracer	Production runs	Average yield (d.c. %)	Specific activity mCi/μmol@EOS	Run time^a^ (min)	Method
[^18^F]ISO-1	50	40 ± 5	1000–14,000	60	One vessel, one step, HPLC, SPE
[^18^F]FTT	40	52 ± 3	1000–35,304	55	One vessel, one step, HPLC, SPE
[^18^F]FTP	12	11 ± 2	2500–4680	60	One vessel, one step, HPLC, SPE
[^18^F]Fallypride	3	68 ± 2	2380–3200	45	One vessel, one step, HPLC, SPE
[^18^F]Flubatine	2	30 ± 1	3119–3220	60	One vessel, two steps, HPLC, SPE
[^18^F]F-Gln	25	11 ± 3	>40^b^	98	Two vessels, two steps, HPLC, SPE

^a^Run time was defined from start of synthesis (radioactivity measured on QMA after radioactivity delivery) to end of synthesis (product formulated in final product vial), including drying [^18^F]F^-^, reaction, purification and formulation

Starting activity ranged from 30 mCi to 1500 mCi; with 30–50 mCi for the developmental runs and 500–1500 mCi for clinical productions

^b^Based on the limit of quantitation from UV spectra

### Synthesis of [^18^F]ISO-1

The one-pot synthesis of [^18^F]ISO-1 was performed in AllinOne module for animal and clinical studies. [^18^F]F^-^ in O-18 water was delivered from cyclotron to the module and trapped by passing the solution through a preconditioned QMA Carb cartridge. Release of [^18^F]F^-^ from QMA Carb cartridge to the reaction vessel was achieved by eluting with a 1 mL basic eluent (0.85 mL MeCN and 0.15 mL water containing 7 mg K222 and 2 mg K_2_CO_3_). Subsequent drying of the solution was conducted at 100 °C for 2 min under vacuum. Anhydrous MeCN (1 mL) was then added to the reaction vessel for azeotropic removal of residual water.

The literature reported method used microwave as the means for radiolabeling. The Trasis module has two conventional heating chambers. Therefore, the radiolabeling method has to been changed to thermo heating. This change also resulted in consistent radiolabeling yields. The mesylate precursor (1.5 mg) in 0.8 mL DMSO was added into the reaction vessel containing dried [^18^F]F^-^, K_2_CO_3_ and K222. The reaction mixture was heated at 100 °C for 10 min and then quenched with 3 mL mobile phase. The reaction mixture was passed through an Al-N Plus cartridge before transfer to HPLC loop. The cartridge was washed with an additional 3 mL of water. Unreacted [^18^F]F^-^ was trapped on an Al-N Plus cartridge and the crude mixture was purified by a semi-preparative HPLC with an Agilent SB-C18 column (5 μm, 100 × 9.4 mm). The mobile phase was 39 % MeOH in 0.1 M NH_4_HCO_2_ buffer at a flow rate of 5 mL/min. The desired product was eluted at approximately 25 min and the fraction (~10 mL) was collected in a 20 mL syringe and then diluted with water. The mixture was passed through a C18 Plus cartridge and rinsed with 10 mL water. The product was eluted with 1.5 mL ethanol and passed through a 0.2 μm sterile Millex® FG filter into final product vial. The final formulation was prepared by adding 15 mL saline into the final product vial.

### Synthesis of [^18^F]FTT

[^18^F]FTT was synthesized on the AllinOne using a similar procedure as described for [^18^F]ISO-1. The precursor that was initially reported was the mesylate precursor. It was found that this precursor gave about 5 % decay-corrected yield. To increase the radiolabeling yield, tosylate precursor was synthesized and radiolabeled. The average yield for tosylate precursor is around 50 % and was then used for the later studies.

The precursor solution (0.8 mg in 0.8 ml DMF) was added to the dried [^18^F]F^-^/K222 in the reaction vessel and the solution was heated at 105 °C for 10 min. After cooling to ambient temperature and removing unreacted [^18^F]F^-^ with an Al-N Plus cartridge, the reaction mixture was purified by a semi-preparative HPLC using an Agilent SB-C18 column (5 μm, 100 × 9.4 mm) and eluting with a mobile phase of 17 % MeCN in 20 mM NH_4_HCO_3_ solution at 5 ml/min flow rate. The purified product was reformulated in the final product vial with saline containing ≤ 10 % ethanol.

### Synthesis of [^18^F]FTP

[^18^F]FTP was synthesized on the AllinOne using a similar procedure as [^18^F]ISO-1. After drying of [^18^F]F^-^, a aliquot of DMSO was added to dry [^18^F]F^-^/K222/K_2_CO_3_ complex prior to the addition of [^18^F]FTP precursor during the manual radiolabeling. The same process cannot be achieved with an automated module. Therefore, another radiolabeling solvent, MeCN, was tested and gave satisfactory radiolabeling results. The mesylate precursor (1.5 mg) was dissolved in 0.8 mL of MeCN. The radiolabeling was carried out at 100 °C for 10 min. The semi-preparative HPLC purification was conducted on Phenomenex Kinetex® 5 μm 150 × 10 mm column and mobile phase of 40 % MeCN in 20 mM NH_4_HCO_3_ with flow rate of 5 mL/min. The collected product was trapped on a ^t^C_18_ Sep-Pak cartridge and the final product was formulated with ≤ 10 % ethanol in saline.

### Synthesis of [^18^F]fallypride

The synthesis of [^18^F]fallypride was adapted from literature with adjustment on the volume of radiolabeling solvent (Mukherjee et al., 2002). The overall process is similar to the process used for [^18^F]ISO-1. The tosylated precursor (4 mg) in 1 mL MeCN was added to the dried [^18^F]F^-^complex and heated at 100 °C for 10 min for radiolabeling. After cooling to room temperature, the reaction mixture was quenched with mobile phase and passed through an Al-N Plus cartridge to remove unreacted [^18^F]F^-^ prior to injection onto a semi-preparative HPLC for purification. Phenomenex Luna C18 (2) column (5 μm, 250 × 10 mm) with mobile phase of 60 % MeCN/40 % 0.1 N NaHCO_3_ in water with a flow rate of 5 mL/min was used. The collected product was enriched and rinsed in ^t^C18 Sep-Pak cartridge and the final product was formulated with ≤10 % ethanol in saline.

### Synthesis of [^18^F]flubatine

The one-vessel, two-step synthesis of [^18^F]flubatine was achieved by displacing the quaternary ammonium leaving group on the precursor with [^18^F]F^-^ and then hydrolyzing N-Boc group. The only optimization of this radiotracer to an automated process was to adjust the volume of radiolabeling solvent. The solution of flubatine precursor (1 mg) in DMSO (0.6 mL) was added to the dried [^18^F]F^-^ complex and heated at 100 °C for 10 min. The reaction mixture was cooled to 80 °C, 1 N HCl solution (1 mL) was the mixture heated at that temperature for 4 min to complete the deprotection step. After cooling to 50 °C, the reaction mixture was neutralized with 1 N NaOH solution (1 mL) and purified via semi-preparative HPLC with a Waters Sunfire column (5 μm, 250 × 10 mm) and mobile phase of 4 % ethanol in PBS buffer at 5 mL/min flow rate was used. The collected product was ready for use after sterile filtration.

### Synthesis of [^18^F]F-Gln

The synthesis of [^18^F]F-Gln, following the route published by Qu et al. (Qu et al., 2010), was developed on the Trasis AllinOne module as a two-vessel, two-step automated procedure. The first step was radiolabeling the precursor and the second step involved removal of the three protecting groups. We employed the published radiolabeling process but devoted some effort to find the optimal drying condition for [^18^F]F^-^ , and [^18^F]F-Gln intermediate . In our experience, the dryness of these two steps is essential to a successful radiolabeling as this is a moisture-sensitive reaction.

After eluting [^18^F]F^-^ from the QMA cartridge, a mild phase-transfer reagent (18-crown-6/KHCO_3_) was added and the [^18^F]F^-^ was dried at 90 °C for 10 min. Precursor (~10 mg) in 1 mL of MeCN was added into the first reaction vessel and the radiolabeling was carried out at 85 °C for 20 min. The intermediate was purified by a semi-prep HPLC system equipped with a Phenomenex Luna C18 column and mobile phase of 55 % MeCN in water at a flow rate of 5 mL/min. The intermediate was collected and passed through a pre-conditioned C18 cartridge to concentrate the radioactivity, which was then eluted with ether and passed through a dry cartridge to the second reaction vessel for the deprotection step. One mL of TFA and 10 μL of anisole were added to the reaction vessel followed by heating at 70 °C for 5 min. The TFA and anisole were removed under nitrogen flow and vacuum. The crude product was dissolved in ether and the mixture was trapped by passing through a Silica Plus cartridge. The final product, [^18^F]F-Gln, was eluted with saline into the final product vial.

### Quality control results

Limited quality control (QC) testing, including testing for pH, radiochemical and chemical purity and identity by HPLC, and residual solvent testing by GC, were performed on [^18^F]fallypride, [^18^F]flubatine, and [^18^F]F-GLN for preclinical studies. Other radiotracers, such as [^18^F]ISO-1, [^18^F]FTT, [^18^F]FTP, had to pass a more rigorous set of QC specifications prior to release for clinical research studies. We followed relevant regulatory guidances, such as USP Chapter 823 and the FDA 21 CFR Part 212. The tests included filter integrity test via bubble test, appearance via visual test, pH test via pH paper, radionuclide identity and purity test via half-life and energy spectrum, radiochemical purity and identity test via analytical HPLC, chemical purity via HPLC, residual solvent analysis via GC, K222 spot test, pyrogenicity, and sterility. An example of QC specifications is listed in Table 2.

**Table 2:** Specifications of ISO-1 for clinical studies

Test	Acceptance criteria
Filter membrane integrity test	Following manufacturer’s specification
pH	4.5–7.5
Appearance	Clear, colorless, and particle-free
Strength (@EOS)	4–14 mCi/mL
Radionuclide identity (dose calibrator)	105.0 min to 115.0 min
Radionuclidic purity (annual test)	≥99.5 % of phantoms
Radiochemical purity	≥90 %
Radiochemical identity	RT difference of radioactivity and reference peaks ≤ 10 %
Drug mass	≤10 μg/ injection dose
Chemical impurity	≤10 μg/ injection dose
Kryptofix	≤50 μg/mL
Bacterial Endotoxin	≤175 EU/ total dose
Residue solvent: MeCN	≤0.41 mg/mL (4.1 mg/day max)
Residue solvent: MeOH	≤3 mg/mL (30 mg/day max)
Residue solvent: EtOH	≤100 mg/mL
Residue solvent: DMSO	≤5 mg/mL (50 mg/day max)
Sterility (results after 14 days)	Sterile (no visible growth)

## Results and Discussion

Six ^18^F-labeled radiopharmaceuticals were produced with full automation on the Trasis AllinOne module without any hardware modification. The processes gave good yields, product purities and specific activities of the final products. The summary of labeling method, decay-corrected radiochemical yield, specific activity at EOS and run time of all six compounds is listed in Table 1.

**Fig. 6:**
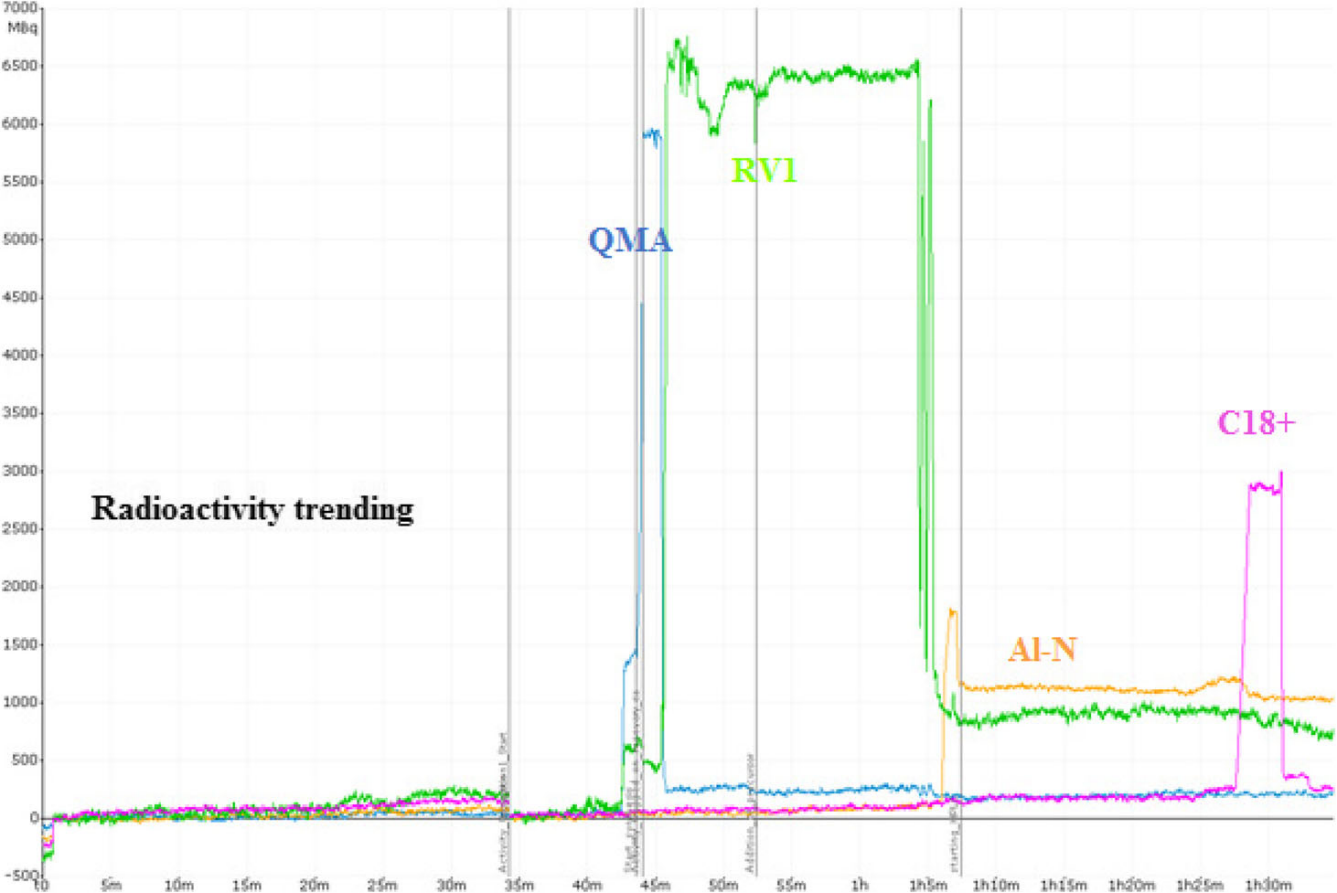
Radioactivity trending of [^18^F]ISO-1

**Fig. 7:**
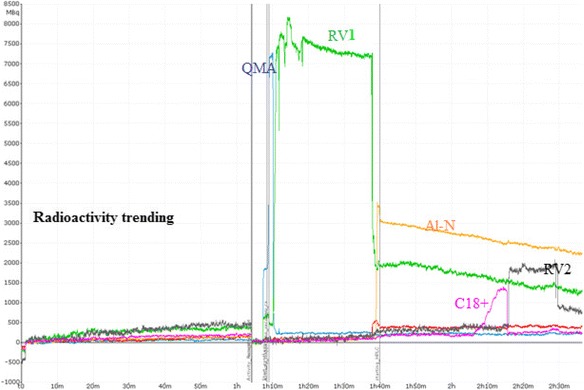
Radioactivity trending of [^18^F]F-Gln

The Trasis AllinOne module is an easy-to-use and versatile module with user-friendly software and interface. Complicated labeling methods requiring two reaction vessels and multiple Sep-Pak purifications can be conducted on it, such as [^18^F]FDopa and [^18^F]F-Gln. There are multiple built-in radioactivity detectors in the AllinOne module so one can track the trending of radioactivity over time. Radioactivity trending for [^18^F]ISO-1 synthesis is shown in Fig. 6 and the radioactivity trending for [^18^F]F-Gln synthesis is shown in Fig. 7. With simultaneous trending, it is easy to check the results of parameter optimization during method development as well as identifying errors when deviations occur during the synthesis. Other trend tracks, such as pressure, temperature, and flow rate are also useful for method development.

For this module, the UV detector operates at a fixed wavelength of 254 nm. Hence, the HPLC is good for production but not preferable for method development. A tunable UV detector would improve the application range of the module. In addition, replacing the flat-bottom reaction vessel with a conical or V-shape reaction vessel would make it possible to reduce the solvent volume and improve the module performance.

**Fig. 8:**
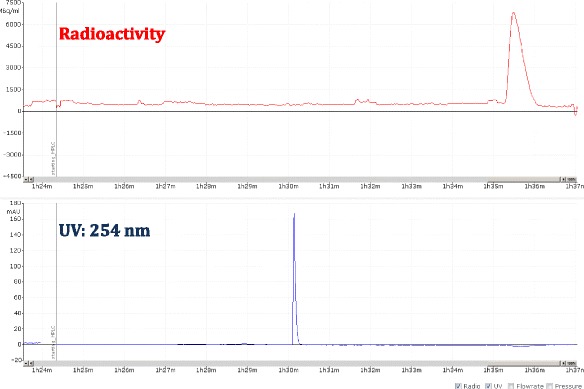
A view of typical semi-preparative chromatogram of [^18^F]fubatine

**Fig. 9:**
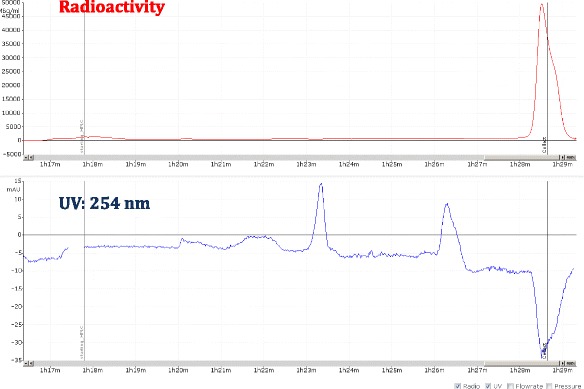
A view of typical semi-preparative chromatogram of [^18^F]fallypride

**Fig. 10:**
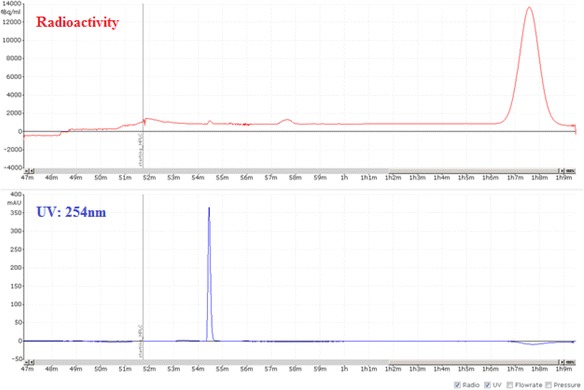
A view of typical semi-preparative chromatogram of [^18^F]FTT

**Fig. 11:**
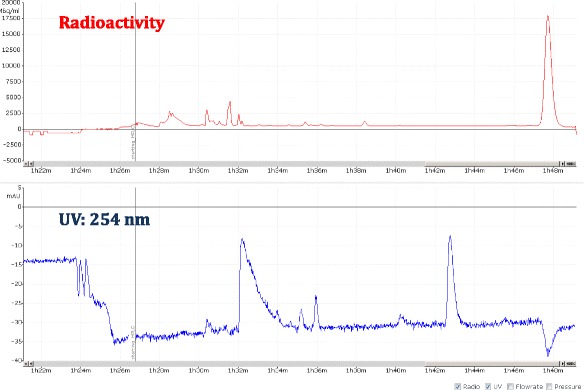
A view of typical semi-preparative chromatogram of [^18^F]FTP

**Fig. 12:**
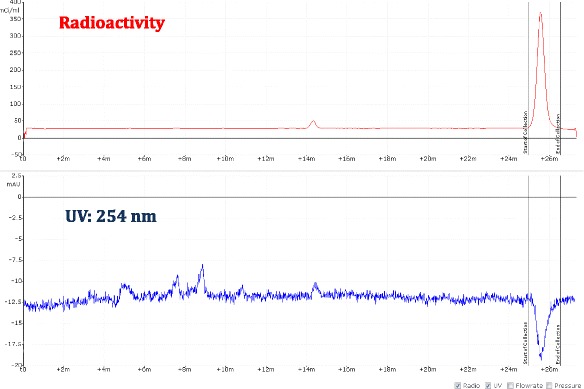
A view of typical semi-preparative chromatogram of [^18^F]F-Gln

**Fig. 13:**
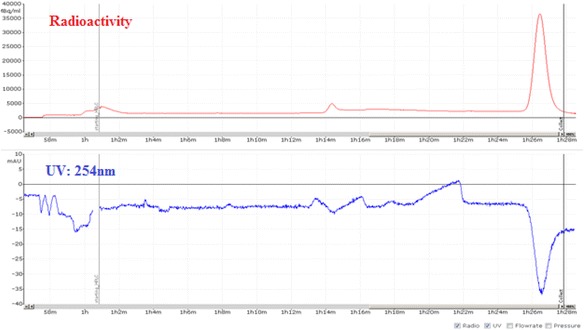
A view of typical semi-preparative chromatogram of [^18^F]ISO-1

**Fig. 14:**
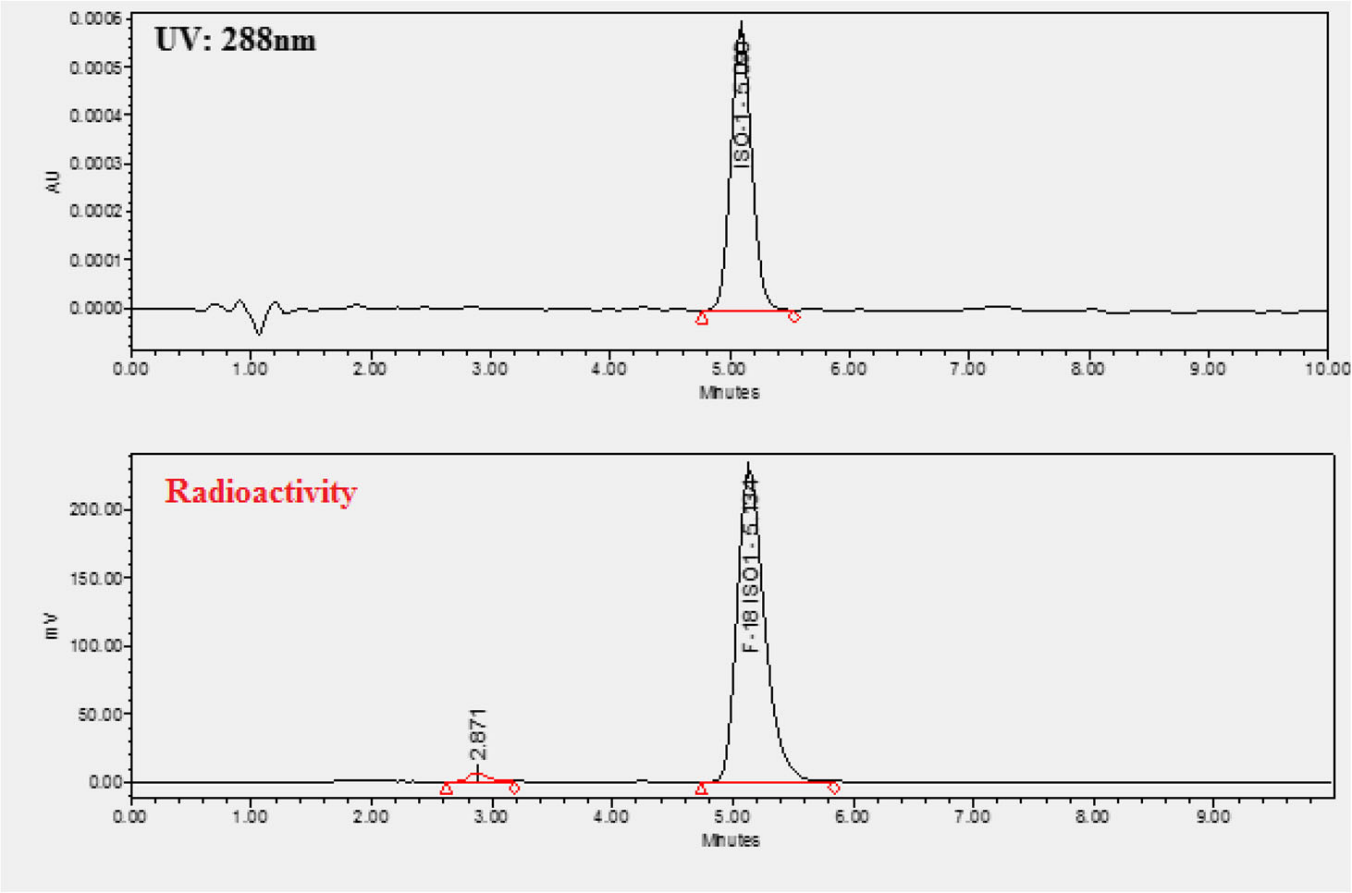
A view of typical analytical chromatogram of [^18^F]ISO-1

Another challenge of the module is the limitation on the syringe used for HPLC collection. The collected HPLC fraction can only be diluted with water into a 20 mL syringe before loading the sample on a C18 cartridge. Therefore, methods for semi-preparative HPLC must be well developed to make sure the trapping on the cartridge is sufficient. The semi-preparative HPLC method of [^18^F]flubatine has been optimized from literature, with the chromatogram shown in Fig. 8; semi-prep HPLC methods for [^18^F]ISO-1, [^18^F]FTT, [^18^F]FTP, and [^18^F]fallypride have also been developed to incorporate solid phase purification and give good mass purity, as the semi-prep chromatograms showed in Figs. 9, 10, 11 and 12. Figure 13 shows a typical semi-preparative HPLC chromatography of the purification of [^18^F]ISO-1. The typical radiochemical purities for all radiotracers are greater than 97 %, except for [^18^F]F-Gln (purity ≥ 90 %). A typical [^18^F]ISO-1 analytical HPLC chromatogram is shown in Fig. 14, demonstrating the high radiochemical and chemical purities.

In addition to semi-prep HPLC method development for the module, Sep-Pak purification has been also done for radiotracer production. The synthesis and purification of [^18^F]F-Gln is more complicated than the other radiotracers described in this study, requiring more than the typical semi-preparative purification and Sep-Pak isolation/elution. Two additional solid phase purifications were introduced in the process. After the initial radiolabeling step and prior to deprotection, the intermediate was isolated and eluted by ether and passage through a Dry Sodium Sulfate Plus Long cartridge to remove residual moisture. Compared to ethanol, ether has a lower boiling point and is easily removed by evaporation. In contrast to the literature method in which the direct formulation was done in the second reaction vessel, further purification of the final [^18^F]F-Gln product was enhanced by introducing a silica cartridge to purify the reaction mixture after deprotection. The final product was trapped on a Silica cartridge to allow for the removal of anisole, TFA and other chemical impurities. The product was eluted with ether and the ether residue was then removed by nitrogen flow under vacuum. The final product was formulated with normal saline or phosphate buffered saline.

**Fig. 15:**
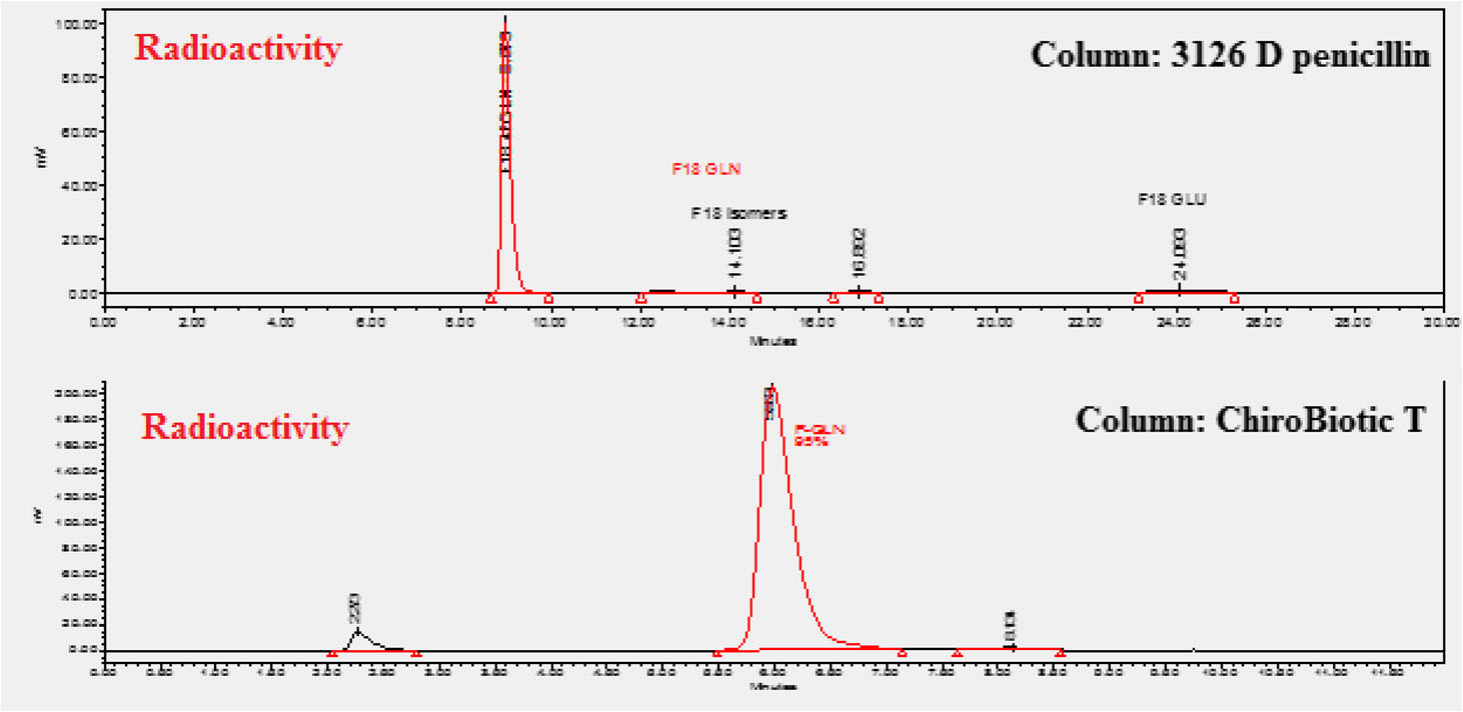
Analytical Radio-HPLC chromatography of [^18^F]F-Gln with different chiral columns

Typical [^18^F]F-Gln analytical HPLC chromatograms with two different conditions are shown in Fig. 15. It was found that the method with Astec CHIROBIOTIC™ T Chiral HPLC column can be used for both radiochemical purity and enantiomer purity. It provides both radiochemical purity and enantiomeric purity with shorter run time than the literature method (Qu et al., 2010). Furthermore, this method works well for metabolism studies for [^18^F]F-Gln.

## Conclusion

We have conducted the fully automated synthesis of six ^18^F-labeled PET tracers on the AllinOne synthesis module without hardware reconfiguration. All tracers were produced in radiochemical yield and with a run time compatible with that required for routine production for PET imaging studies. Three radiotracers are currently being used in clinical research studies using the methods described in this report.

## Abbreviations

cGMP: Current good manufacturing practice
DMF: Dimethylformamide
DMSO: Dimethyl Sulfoxide
EOS: End of Synthesis
FDG: Fluorodexoglucose
FGln: (*2S, 4R*)-4-Fluoroglutamine
FTP: LS-3134 or Flurotripride
GC: Gas Chromatography
HPLC: High Pressure Liquid Chromatography
MeCN: Acetonitrile
PARP1 or FTT: FluorThanatracer
PET: Position Emission Tomography
QC: Quality control
RV: Reaction vessel
SPE: Solid Phase Extraction
TFA: Trifluoroacetic Acid
USP: United States Pharmacopeia
UV: Ultraviolet
